# Volumetric Analysis of Motor Cortex and Basal Ganglia in Pediatric Celiac Disease Patients Using volBrain: Implications for Neurological Dysfunction-Preliminary Results

**DOI:** 10.3390/diagnostics14222559

**Published:** 2024-11-14

**Authors:** Filip Murn, Lana Loncar, Jasna Lenicek Krleza, Goran Roic, Iva Hojsak, Zrinjka Misak, Ana Tripalo Batos

**Affiliations:** 1 Department of Radiology, Children’s Hospital Zagreb, 10000 Zagreb, Croatia; murn.filip@gmail.com (F.M.); goran.roic@kdb.hr (G.R.); ana.tripalobatos@kdb.hr (A.T.B.); 2 Department of Neuropediatrics, Children’s Hospital Zagreb, 10000 Zagreb, Croatia; lana.loncar@kdb.hr; 3 Department of Laboratory Diagnostics, Children’s Hospital Zagreb, 10000 Zagreb, Croatia; 4 University Department of Nursing, Catholic University of Croatia, Ilica 244, 10000 Zagreb, Croatia; 5 Department of Laboratory Medical Diagnostics, University of Applied Health Sciences Zagreb, 10000 Zagreb, Croatia; 6 Referral Center for Pediatric Gastroenterology and Nutrition, Children’s Hospital Zagreb, 10000 Zagreb, Croatia; ivahojsak@gmail.com (I.H.); zrinjka.misak@gmail.com (Z.M.); 7 School of Medicine, University of Zagreb, 10000 Zagreb, Croatia; 8 School of Medicine, University J. J. Strossmayer, 31000 Osijek, Croatia

**Keywords:** celiac disease, neurological manifestation, MRI volumetry

## Abstract

Background/Objectives: Celiac disease (CD) is a common immune-mediated, chronic systemic disorder that is treated with a strict, life-long gluten-free diet (GFD). In addition to gastrointestinal manifestations, CD also presents with a variety of extraintestinal symptoms, including significant neurological and neuropsychiatric symptoms. Among these neurological manifestations, motor dysfunctions are particularly notable. The aim of this study is to investigate the potential volumetric differences in brain structures, particularly the motor cortex and basal ganglia, between pediatric CD patients and healthy controls using the volBrain software AssemblyNet version 1.0. Methods: This prospective study included pediatric patients with CD who complained of neurological symptoms and were scheduled for brain magnetic resonance imaging (MRI). All children had been previously diagnosed with CD and their adherence to GFD was evaluated using the Biagi score. Brain MRIs were performed on all included patients to obtain volumetry at the onset of the disease. For volumetric and segmentation data, the volBrain software was used. Results: In total, 12 pediatric patients with CD were included, with a median duration of a GFD of 5.3 years at the time of the MRI examination. There were no statistically significant differences between patients compliant with the GFD and those non-compliant in terms of age or duration of GFD. Volumetric analysis revealed deviations in all patients analyzed, which involved either a decrease or increase in the volume of the structures studied. Conclusion: Despite the limited number of patients in this study, the initial findings support previously described neurological manifestations in patients with CD. Newly developed MRI tools have the potential to enable a more detailed analysis of disease progression and its impact on the motor cortex.

## 1. Introduction

Celiac disease (CD) is an immune-mediated disorder triggered by gluten intake in genetically predisposed individuals [1]. The overall prevalence of CD in the general population ranges from 0.5% to 2%, with an average of about 1% [2]. CD is characterized by the presence of a variable combination of gluten-dependent clinical manifestations, CD-specific antibodies (autoantibodies against tissue transglutaminase, endomysial antibodies, and antibodies against deamidated forms of gliadin peptides), human leukocyte antigen (HLA)-DQ2 or HLA-DQ8 haplotypes, and enteropathy. The ingestion of gluten in predisposed individuals may initiate the pathophysiological cascade of events including adaptive and innate immune response to gluten proteins and leads to the development of the coeliac autoimmunity and enteropathy with a significant role in the pathogenesis played by the HLA-restricted gliadin-specific intestinal T cell response [2,3]. Traditionally recognized for its gastrointestinal symptoms, CD also presents with a variety of extraintestinal manifestations, including significant neurological and neuropsychiatric symptoms [4,5].

The exact pathophysiology of the neurological symptoms in CD remains unclear, though autoimmune and inflammatory mechanisms are suspected to play a role. Additionally, nutrient deficiencies resulting from malabsorption, such as vitamin B6 deficiency, have been implicated in the neurological and psychiatric symptoms [6]. The frequency of extraintestinal symptoms increases with age. An Italian study from 2021 showed that the frequency of neurological symptoms in children with celiac disease has increased from 10 to 24% in the past 30 years [7]. The most common neurological symptoms associated with celiac disease are ataxia, peripheral neuropathy, headache/migraine, epilepsy, attention deficit hyperactivity disorder (ADHD) and other psychiatric disorders, and autism [5,8]. These symptoms may arise as complications or initial presentations of CD, with the research indicating that neurological symptoms can sometimes be primary indicators of the disease, even in the absence of gastrointestinal manifestations [1,4]. The distribution of certain neurological symptoms differs between age groups. In the adult population, motor dysfunctions are particularly notable among these neurological manifestations. Studies using transcranial magnetic stimulation (TMS) have shown that CD patients, even those without overt neurological symptoms, exhibit significant intracortical and interhemispheric motor disinhibition [4,9].

Further evidence indicates that newly diagnosed adult CD patients already exhibit detectable changes in brain MRI scans, especially on a 1 mm slice thickness sagittal 3D T1 weighted image which is the best image for analyzing the brain anatomy. These findings highlight the importance of early diagnosis and strict adherence to a gluten-free diet (GFD) to potentially mitigate neurological complications and improve patient outcomes. However, it remains unknown whether a GFD can reverse or significantly alter the progression of existing neurological issues [10].

### Brain Motor Network

Motor learning and the consolidation of new motor skills depend on plasticity within the motor cortex and striatum, which are essential regions for motor control. A broad network encompassing the basal ganglia, cerebellum, motor cortex, and brainstem facilitates motor learning by selecting and executing motor programs. The basal ganglia (BG) consist of several nuclei, including the caudate nucleus (CN), putamen (striatum), globus pallidus (GP), substantia nigra, and subthalamic nucleus (STN) [11]. These structures form cortico–BG–thalamic loops that modulate thalamic activity and influence cortical processing. The basal ganglia are critical for action selection, integrating sensory evidence, and disinhibiting appropriate action plans. The cerebellum ensures motor accuracy, particularly during adaptation to environmental changes [12]. Damage to the cerebellum impairs motor adaptation, underscoring its role in motor learning as confirmed by imaging studies. The cortico–basal ganglia loop, involving the motor cortex, striatum, substantia nigra pars reticulata, thalamus, and motor cortex, selects concrete actions through dopamine-modulated Hebbian learning. This closed loop forms goal-response maps that link objectives to appropriate actions. Learning is triggered by a phasic increase in dopamine upon detecting novel movements, inducing plasticity in striatal neurons [12,13]. The prefrontal cortex (PFC) is a complex brain region essential for higher order functions such as working memory, abstraction, sensory attention, value-based decision-making, planning, and motor control. The PFC’s ability to generate persistent internal representations that organize behavior over time is fundamental to its role in cognitive processes. Damage to the PFC can result in significant functional impairments, underscoring its critical importance in human behavior and cognition. Motor-related cortical areas are crucial for both action generation and perception [14]. Research has identified motor complexity-sensitive regions including the pars opercularis of the inferior frontal gyrus (IFG), ventral premotor cortex (PMv), primary motor cortex (M1), inferior parietal lobule (IPL), and middle occipital gyrus (MOG) during action execution. During action observation, activity was noted in the pars opercularis IFG/PMv and M1. These findings highlight the involvement of these areas in processing motor complexity during both action perception and execution [15,16]. The primary motor cortex (M1), located in the precentral gyrus of the frontal lobe, is essential for executing voluntary movements. The prefrontal cortex (PFC) is a large and complex region associated with numerous higher cognitive processes. The premotor cortex (PMC) regulates higher level motor plans by potentiating or depotentiating specific motor actions, supported by preparatory activity studies for movements and decisions. The orbitofrontal cortex (OFC), at the apex of the prefrontal hierarchy, integrates sensory, emotional, and cognitive inputs, optimizing goal-selection policies. The OFC’s role in evaluating and selecting goals is crucial for complex decision-making processes, underscoring its fundamental premotor function [17].

The aim of this study is to investigate the potential volumetric differences in brain structures, particularly the motor cortex and basal ganglia, between pediatric CD patients and healthy controls using the volBrain software.

## 2. Materials and Methods

This is a prospective study approved by the Ethics Committee of the Children’s Hospital Zagreb. This study included pediatric patients with CD who complained of neurological symptoms and were therefore scheduled for brain magnetic resonance imaging (MRI). All children had been previously diagnosed with CD in the Children’s Hospital Zagreb according to the European Society for Paediatric Gastroenterology, Hepatology and Nutrition guidelines [1]. The diagnosis of celiac disease was made on the basis of general clinical symptoms, blood tests, and tissue samples. The antibodies specific for celiac disease used in the diagnosis are antibodies to tissue transglutaminase (anti-tTG), endomysial antibodies (EMA), and antibodies to deamidated gliadin peptide (DGP). These antibodies can belong to the immunoglobulin (Ig) A and G class, but only those of the IgA class are considered highly sensitive and specific for celiac disease. IgG class antibodies have a high percentage of false positives, so their use is limited to individuals with selective IgA deficiency.

Therefore, in our study, as an initial test in children with suspected celiac disease, total IgA in serum (immunoturbidimetric method, Beckman Coulter analyzer AU680; Mishima Factory and Laboratory, Tokyo, Japan) and IgA class antibodies to tissue transglutaminase (anti-tTG-IgA) were determined (EliA test—fluorescent enzyme immunoassay on the Thermo Fisher Phadia 200 analyzer; Uppsala, Sweden). In children with a low concentration of total IgA in the serum (IgA deficiency), the determination of IgG class antibodies to the deamidated gliadin peptide was carried out in a second step using the DGP-IgG (EliA test—fluorescent enzyme immunoassay on the ThermoFisher Phadia 200 analyzer) and/or endomysial antibodies using the EMA (indirect immunofluorescence, immunofluorescence microscope, EurospItal diagnostic reagent kit).

The further course of the diagnostic procedure depended on the antibody titer. High levels of anti-tTG-IgA that are ten times higher than the upper limit of normal and positive EMA test were used as a criterion for choosing a diagnostic procedure without biopsy according to the algorithm as a good predictor of celiac enteropathy (Marsh 2–3) [1].

At the regular follow-up visit at the pediatric gastroenterology clinic, all children with CD were asked whether they had any of the following neurological symptoms: headache, dizziness, mood swings, paresthesias, epilepsy, tinnitus, visual or hearing problems, syncope, and tremor. At the same visit, adherence to the gluten-free diet (GFD) was evaluated by Biagi score; patients with Biagi scores 0-I do not follow a strict GFD, with a score of II indicating that patients follow a GFD but with errors that require correction, and scores of III-IV indicating that patients follow a strict GFD [18]. If any neurological symptom was present, the child was scheduled for brain MRI.

All included patients underwent brain magnetic resonance imaging (MRI) on our two MRI devices, one with 1.5T and the other with 3T, to obtain volumetry at the onset of the disease. The standard MR brain protocol included sagittal 3D high-resolution T1-weighted images (slice thickness 1 mm); axial and coronal T2-weighted images, axial balanced steady state free precession line acquisition with undersampling (BLADE), axial T2 gradient echo (GRE), or susceptibility weighted imaging (SWI), diffusion-weighted imaging (DWI)/apparent diffusion coefficient (ADC). The 1 mm slice thick sagittal 3D T1 weighted image is particularly important because of its thickness and isovolumetric characteristics as the best sequence for analyzing brain anatomy and volume. A standard brain MRI protocol was used and included multiple sequences to capture detailed images of the brain’s structure and identify any abnormalities. The used sequences in this protocol are as follows:

1. Sagittal 3D T1-Weighted (T1W) Sequence: This sequence is crucial for high-resolution anatomical imaging, especially with a 1 mm slice thickness. The thin, isovolumetric slices allow for excellent spatial resolution and make it easier to analyze brain anatomy and volume. The 3D T1W images are ideal for evaluating the brain’s structural details and are particularly helpful in detecting abnormalities in the cortex and white matter.
2. Axial and Coronal T2-Weighted (T2W) Sequences: These sequences provide contrast between different types of brain tissues, making it easier to spot lesions, edema, or inflammation. T2W imaging is essential for identifying pathologies like tumors, multiple sclerosis lesions, and areas affected by ischemia.
3. Axial BLADE Sequence: This is a motion-compensated sequence, useful for reducing artifacts in patients who may have difficulty remaining still. It helps produce clearer images in cases where patient motion might otherwise degrade image quality.
4. Axial T2 GRE or SWI: These sequences are highly sensitive to magnetic susceptibility effects, making them effective for detecting small hemorrhages, calcifications, and vascular malformations. SWI, in particular, is beneficial for evaluating microbleeds and iron deposits.
5. DWI and ADC: These sequences are critical for identifying acute ischemic strokes, as they can show changes in water diffusion within minutes of a stroke onset. DWI combined with ADC mapping helps differentiate between acute and chronic ischemic lesions.

Together, these sequences provide a comprehensive view of brain structure, pathology, and function. The 1 mm slice thickness in the 3D T1W sequence is especially valuable for precise anatomical assessment, allowing for multiplanar reconstructions and volume measurements of brain regions. This combination of sequences enhances diagnostic accuracy across a wide range of neurological conditions.

Volumetric analysis was performed using volBrain software from 3D high-resolution T1-weighted images. The same standardized brain imaging protocol was used regardless of the magnetic field strength (1.5T or 3T). The quality of the 3D T1-weighted sequence for volume calculation was verified by the volBrain software. The MRI scans were reviewed by three neuroradiologists. Exclusion criteria for this study included the presence of any expansive processes, ischemia (acute or chronic), or non-neoplastic lesions, such as arachnoid cysts.

### 2.1. MRI Volumetry

**Figure 1:**
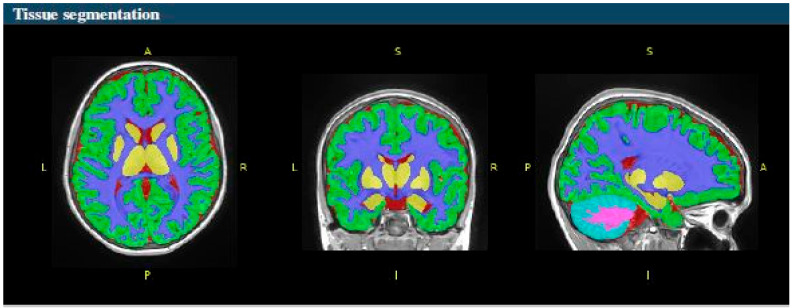
The 3D T1-weighted image tissue segmentation in axial (**left image**), coronal (**middle image**), and sagittal plane (**right image**); A—anterior; P—posterior; R—right; L—left; S—superior; I—inferior; Red: cerebrospinal fluid (CSF); Green: cortical gray matter (GM); Blue: white matter (WM); Yellow: basal ganglia (BG); Turquoise: cerebellum GM; Pink: cerebellum WM.

**Figure 2:**
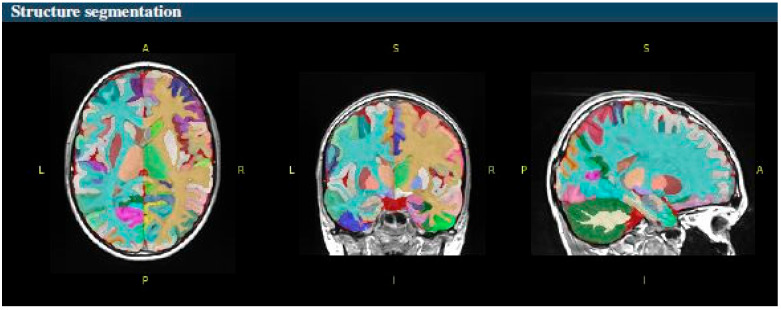
3D T1-weighted image structure segmentation in axial (**left image**), coronal (**middle image**), and sagittal plane (**right image**); A—anterior; P—posterior; R—-right; L—left; S—superior; I—inferior. Color maps for brain segmentation are used from Mindboggle software https://github.com/nipy/mindboggle/blob/master/mindboggle/mio/colors.py (accessed on 20 October 2024).

The volBrain software system provides volumetric and segmentation data (Figure 1), including asymmetry ratios, at various scales. It measures the intracranial cavity (ICC), which is the sum of white matter (WM), gray matter (GM), and cerebrospinal fluid (CSF). Additionally, it provides tissue volumes of WM, GM, and CSF, as well as the volumes of the cerebrum, cerebellum, and brainstem, including the left and right cerebrum and the cerebellum. The system also measures the volumes of lateral ventricles and subcortical gray matter structures such as the putamen, caudate, pallidum, thalamus, hippocampus, amygdala, and accumbens (Figure 2).

In this study, we used volBrain software as its primary advantage lies in its remarkable computational efficiency. The entire pipeline takes an average of only 12 min to complete, covering the following critical steps like:

○ Denoising (30 s);
○ Inhomogeneity correction (30 s);
○ Registration into MNI space (2 min);
○ Fine inhomogeneity correction using SPM (3 min);
○ Brain extraction (2 min);
○ Structure labeling (3 min).

The system operates through a simple, intuitive web interface that allows users to submit MRI data, and in a matter of minutes, receive detailed volumetric analyses in both PDF and CSV formats. Additionally, users can download the segmentation results in either native or MNI space. This Software-as-a-Service (SaaS) model is a crucial innovation, making volBrain widely accessible to users around the globe without complex configurations [19,20].

### 2.2. Pipeline Overview

The volBrain pipeline is a comprehensive image processing workflow that transforms input MRI data into detailed volumetric reports. It consists of the following key steps:

1. Denoising: This step improves the quality of the input images by applying the spatially adaptive non-local means (SANLMs) filter to reduce noise. This filter adapts to varying noise levels across the image, making it ideal for processing MRI data with spatially variable noise [21].
2. Inhomogeneity correction: Inhomogeneities in MRI images are corrected in two phases. First, the N4 method is applied for coarse correction, followed by fine correction using SPM after the image is registered to MNI space [22].
3. Registration to MNI space: for consistent and standardized analysis, volBrain registers images to the Montreal Neurological Institute (MNI152) space using the ANTs software version 1.0. This registration process ensures that all images are spatially normalized, providing a common reference frame for further analysis [23].
4. Tissue classification and structure segmentation: volBrain employs a sophisticated segmentation process based on multi-atlas patch-based label fusion to classify various brain tissues (e.g., white matter, gray matter, cerebrospinal fluid) and segment key brain structures. This non-local label fusion method ensures that the segmentation is both accurate and computationally efficient.
5. Subcortical structure segmentation: volBrain’s subcortical structure segmentation is particularly noteworthy, as it delivers highly accurate and reproducible results for critical structures such as the hippocampus, thalamus, and caudate nucleus. The platform’s unique approach, which includes modifications to the non-local label fusion algorithm, further enhances the quality and consistency of the segmentation results.

### 2.3. Performance Evaluation

In comparison with other well-established MRI analysis tools like FreeSurfer and FIRST, volBrain consistently demonstrates superior performance across various evaluation metrics, including the following:

1. Dice coefficient: In terms of segmentation accuracy, volBrain achieves the highest Dice coefficients across multiple structures. For example, volBrain’s Dice score for the hippocampus is 0.953, significantly higher than FreeSurfer’s 0.788 and FIRST’s 0.843. This level of accuracy is critical for clinical and research applications where precise volume measurements are required [24].
2. Volume estimation: When comparing automatic volume estimates with manual segmentation (considered the gold standard), volBrain shows a higher correlation with manual measurements than FreeSurfer or FIRST. This consistency in volume estimation is crucial for ensuring reliable and interpretable results across different subjects.
3. Reproducibility: volBrain’s reproducibility has been rigorously tested on datasets like the OASIS dataset, which includes multiple scans of the same subjects. volBrain’s reproducibility outperforms both FreeSurfer and FIRST, with a significantly lower failure rate. While FIRST encountered issues in 10% of the cases, volBrain maintained a near-perfect success rate, making it highly suitable for clinical applications [20].

Out of all the mentioned other automated software tools for volumetric analysis, due to all the above advantages, we decided on the volBrain software. For the volumetric analysis, we used data that were already automated in the volBrain software from their files for the pediatric population.

## 3. Results

In total, 12 pediatric CD patients were included (6 girls, median age 13.3 years, range 6.9 to 18.5 years). The median duration of GFD (from the diagnosis of CD till brain MRI) was 5.3 years (range 1 to 14.5 years). Four patients (two girls) did not adhere to GFD (all had Biagi I), and the others were compliant with GFD (Biagi III–IV). There were no statistically significant differences between the compliant vs. non-compliant to GFD patients regarding age or duration of GFD (for both *p* > 0.05). Basic characteristics are shown in Table 1.

Volumetric analysis revealed structural deviations in each patient analyzed (Table 2.). Volumetric deviations refer to either a decreased or increased volume in the analyzed structures. In addition to the analysis of volumetric data, information on whether the analyzed patients adhere to the prescribed gluten-free diet is also included. A total of six children (50%, three girls) had decreased volume: one of the frontal lobe, one of the supramarginal gyrus, one of the middle occipital gyrus, and one of the cerebellum, while one patient had decreased volume in the putamen and globus pallidum, and one in the nucleus caudatus, putamen, globus pallidum, opercular inferior frontal gyrus, and middle occipital gyrus. The volume of the putamen, globus pallidum, and middle occipital gyrus was decreased in two patients each. The volume of the cerebellum was decreased in one patient and so was the volume of the opercular inferior frontal gyrus and supramarginal gyrus. None of the patients had a reduced volume of the precentral gyrus, precentral medial gyrus, and angular gyrus. None of the children had any other brain abnormalities detected by MRI.

## 4. Discussion

This is the first prospective study using the volBrain software in children with celiac disease to analyze brain volumetric values. We demonstrated that 50% of patients with a previously known diagnosis of celiac disease and neurological symptoms have a decreased volume of cortex, cerebellum, or basal ganglia. Among adult patients with CD, a range of 10–22.5% for the prevalence of neurologic dysfunctions has been reported. Sixty percent of adult patients with neurologic symptoms are assumed to have structural and morphological brain abnormalities, especially changes in the cortex, cerebellum, and basal ganglia. These changes align with the most frequently reported neurological symptoms—headache, ataxia, and neuropathy [8,25,26,27,28,29]. The prevalence of neurologic symptoms among children with CD is still unclear. Among our cohort of patients, 92% reported having headache. None of our patients had gait, sensory, or any other motor disturbance which are known to be present in the majority of adult CD patients with neurologic problems. Nevertheless, we demonstrated that 50% of our patients have volumetric abnormalities of cortex, cerebellum, and BG. Although the number of analyzed patients is small, the abnormalities we found can correlate with those found in adult patients who also have connected clinical symptoms opposite to children. Similar results can emphasize this group of children as a group that may have progression of neurological symptoms in adulthood. Follow-up of these patients will show whether there is a link between neurological symptoms and MRI findings in childhood and adulthood in this group.

To analyze motor function, it is essential to understand the connections between the BG and the cerebellum. Studies have demonstrated that the BG and cerebellum play complementary roles in motor learning. The basal ganglia are crucial for selecting appropriate motor actions based on novelty and reward signals, while the cerebellum fine-tunes these actions to minimize errors. This division of labor supports the super-learning hypothesis, suggesting that different brain regions employ distinct learning mechanisms to achieve coordinated motor behavior. The model’s ability to replicate human motor adaptation and variability in learning underscores its potential for studying complex motor tasks and neurological disorders [12]. These findings challenge traditional models of BG function, which primarily emphasize action selection and vigor modulation. Instead, results support a model where the BG, particularly the dorsolateral striatum (DLS), play a direct role in encoding and specifying the detailed kinematics of learned motor skills. This function appears to be independent of motor cortex inputs and involves the continuous representation of kinematic variables throughout the learned behavior [30,31]. In addition, the BG contribute to perception, attention, and consciousness by integrating sensory processing, temporal binding, and predictive coding. Understanding these functions offers insights into BG-related pathologies and their effects on cognitive and perceptual processes. The BG are involved in predictive processing, extracting statistical relationships from sensory information to guide behavior [13]. Our findings suggest that motor dysfunctions develop later in the course of the disease related with the duration of gluten exposure and we showed that subclinical CNS manifestations of CD are also present in childhood. We also demonstrated that volumetric changes were present regardless of compliance with GFD. These can relate to the fact that neurologic changes are less responsive to GFD than gastrointestinal or other extraintestinal symptoms. Hadjivassiliou et al. showed in adult patients with CD that achieving serological negativity with a strict GFD is associated with an at-least reduced progression of brain atrophy [32]. However, the effects of GFD on neurological manifestations are still debated in the literature. A new perspective is provided by the recent literature elaborating on the role of inflammation in the natural history of CD, as well as on the potential benefits of anti-inflammatory diets for CD patients [3]. Whether anti-inflammatory diets could also have a protective effect on the risk of developing extraintestinal manifestations remains to be elucidated.

Although the results of this study are preliminary, the finding that 50% of included patients exhibit changes in the volume of at least one part of the motor cortex opens the door to further research on the impact of CD on motor functions. The objective of this study is to conduct a volumetric analysis of pediatric patients with CD who have neurologic symptoms. Conducting a volumetric analysis on CF patients without neurological symptoms and also newly diagnosed patients at the time of diagnosis will be crucial to understanding the impact on the motor cortex. Monitoring patients with reduced volume of the motor cortex and basal ganglia is crucial in maintaining normal motor function in these patients. It enables early intervention at the appearance of the first motor dysfunction symptoms. Monitoring motor cortex volume enables for patients with CD to maintain normal life quality. Therefore, it would also be interesting to compare analyzed neuroimaging parameters of children and adults and correlate those findings with the development of clinical manifestations.

### Limitations of the Study

As previously mentioned, the limitations of this study include the small number of patients, as these are preliminary results. The data on standard volumetric values for each age group, used as reference values, are derived from the pediatric library of the automated volBrain software. Additionally, it is a challenge that the patient’s age must be entered as a full year interval. In our study, we used two MRI devices with different field strengths (1.5T and 3T). A limitation of this approach is that patients scanned with the 3T MRI had higher resolution 3D T1-weighted images, which may allow for more precise brain volumetry analysis using the volBrain software.

## 5. Conclusions

Despite the limited number of patients in this study, the initial findings support previously described neurological manifestations in patients with CD. Using automated volumetric analysis for MRI devices, such as the volBrain software used in our study, there is potential for a more detailed analysis of disease progression in patients with CD who do not strictly adhere to a GFD and its impact on the motor cortex. However, it is important in further investigations to overcome the limitations of this study and perform a targeted neurological examination in comparison to the results of MRI volumetry.

## Figures and Tables

**Table 1:** Basic characteristics of included patients.

	Non-Compliant to GFD (Biagi I) *N* = 4	Compliant to GFD (Biagi III–IV) *N* = 8
Girls (*N*, %)	2 (50%)	4 (50%)
Boys (*N*, %)	2 (50%)	4 (50%)
Median age at the time of MRI (range), years	14.6 (12.7–18.5)	11.5 (6.9–14.8)
Median duration of GFD (range), years	6.6 (1.25–14.4)	4.4 (1–9.9)
Neurological symptoms		
Headache	4 (100%)	7 (87.5%)
Instability	0	0
Paresthesias	0	0
Tremor	0	1 (12.5%)
Syncope	0	1 (12.5%)
Mood swings	1 (12.5%)	0
Epilepsy	0	0
Visual disorders	0	0
Hearing disorder	0	0

**Table 2:** Results of the volumetric analysis.

Age	Sex	Cbl	NC	PTM	GLP	FL	OPINFFG	PG	PMG	AG	SMG	MOG	DC
15	F	N	N	0.558 (0.593, 0.752)	0.200 (0.212, 0.269)	N	N	N	N	N	N	1.160 (0.758, 1.117) L	Y
13	F	N	N	N		N	0.725 (0.454, 0.717)	N	N	N	N	N	N
7	F	N	N	N		N	N	N	N	2.319 (1.631, 2.300) R	N	N	Y
14	F	N	0.201 (0.203, 0.271) R	0.579 (0.593, 0.753)	0.102 (0.103, 0.133)R	N	0.202 (0.207, 0.369) L	N	N	N	N	0.744 (0.776, 1.129)	Y
15	F	N	N	N		14.236 (14.245, 16.105)	N	N	N	N	N	N	N
10	F	N	0.264 (0.200, 0.262) L	N		N	N	N	N	N	1.152 (1.188, 1.701)	N	Y
14	M	N	N	N		8.148 (7.125, 8.129) R	N	N	0.263 (0.165, 0.260) R	N	N	N	N
19	M	11.106 (8.184, 10.247)	N	N		N	N	N	N	N	N	0.349 (0.388, 0.619) R	N
14	F	10.536 (8.215, 10.306)	N	N		N	0.781 (0.434, 0.714)	1.209 (0.923, 1.191) R	N	N	N	N	Y
11	M	N	N	N		16.629 (14.539, 16.366)	0.429 (0.212, 0.400) R	2.309 (1.853, 2.299)	N	N	N	N	Y
10	F	N	N	N	0.139 (0.108, 0.136) L	N	N	N	N	N	N	N	Y
12	M	7.811 (8.257, 10.321)	N	N	N	N	N	N	N	N	N	N	Y

Abbreviations Cbl—cerebellum; NC—nucleus caudatus; PTM—putamen; GLP—globus pallidus; FL—frontal lobe; OPINFFG—opercular inferior frontal gyrus; PG—precentral gyrus; PMG—precentral medial gyrus; AG—angular gyrus; SMG—supramarginal gyrus; MOG—middle occipital gyrus; DC—diet compliance; L—left hemisphere; R—right hemisphere; Y-yes; N in DC column -no; N—normal values; Numbers in brackets represent normal values for that brain region.

## Data Availability

Original data can be obtained from Jasna Lenicek Krleza upon request.
